# Peritoneal Tuberculosis in Infants: Diagnostic Challenges

**DOI:** 10.1055/s-0038-1675406

**Published:** 2018-11-20

**Authors:** Ndeye Aby Ndoye, Papa Alassane Mbaye, Jacques Noel Tendeng, Lissoune Cissé, Mamadou Lamine Diao, Mactar Dieng, Doudou Gueye, Dibor Niang, Oumar Ndour, Gabriel Ngom

**Affiliations:** 1 Department of Pediatric Surgery of Medecine, Universite Cheikh Anta Diop, Dakar, Senegal; 2 Department of Pediatric Surgery, Albert Royer Children's Hospital, Dakar, Senegal; 3 Chirurgie, Universite Gaston Berger, Saint-Louis, Senegal; 4 Chirurgie Viscérale, Regional Hospital of Saint-Louis, Senegal, Saint-Louis, Senegal; 5 Department of Anesthesiology, Regional Hospital of Saint-Louis, Senegal, Saint-Louis, Senegal; 6 Department of Pathologists, Universite Gaston Berger, Saint-Louis, Senegal; 7 Department of Pediatric Surgery, Hopital Aristide Le Dantec, Dakar, Dakar, Senegal

**Keywords:** peritoneal tuberculosis, infant, acute abdomen, laparotomy

## Abstract

Abdominal tuberculosis is rare in immunocompetent infants. We report on two infants with peritoneal tuberculosis (6 and 8 months) who underwent laparotomy for suspected intussusception. In the first patient, characteristic lesions of peritoneal tuberculosis were observed intraoperatively with presence of multiple granulations. Tuberculin intradermal reaction (IDRt) was positive and tuberculous contagium could be cultured. In the second patient, the IDRt and GeneXpert tests were negative. In both patients, the histopathological examination of the biopsy specimens confirmed the diagnosis of peritoneal tuberculosis. The clinical courses under tuberculostatic therapy were favorable in both cases.

## Introduction

Childhood tuberculosis (TB) is a public health problem around the world. According to the World Health Organization (WHO), the percentage of TB in children varies from 3% to over 25%, depending on the country. 1 The abdominal localization comes in fourth position after pulmonary, ganglionic, pleural, and osteoarticular manifestation. 2 If the abdomen is affected, peritoneal TB is the most common, followed by an infection of the intestines. Although in industrialized countries, the frequency of mycobacterium TB-associated infection is mainly related to human immunodeficiency virus; in sub-Saharan Africa, peritoneal TB is not an exceptional case in pediatrics. Nevertheless, even in endemic countries, peritoneal TB remains extremely rare in infants with a predominance in the older child. 3 4 5 We report on two cases with abdominal TB and discuss the diagnostic difficulties related to this localization in young children.

## Observations

### Case 1

**Fig. 1:**
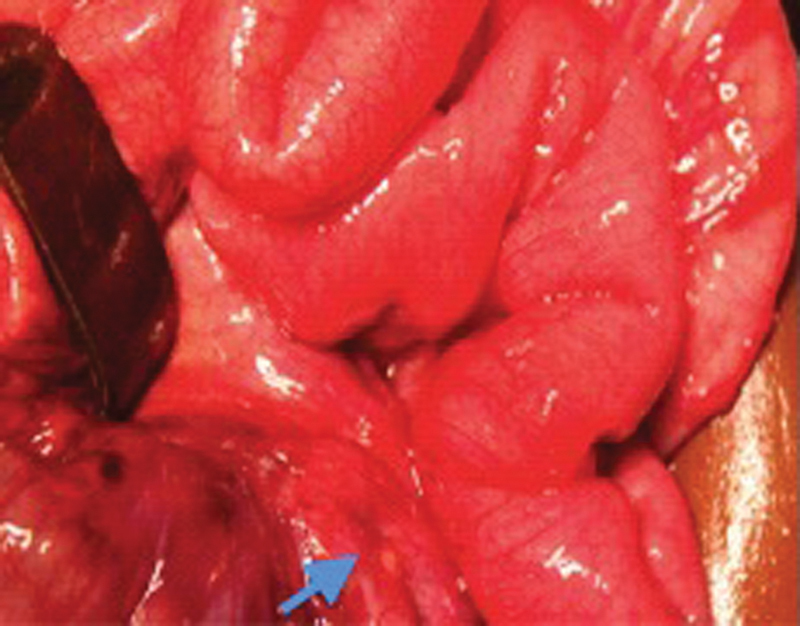
Granulations on the ileum (see arrow).

**Fig. 2:**
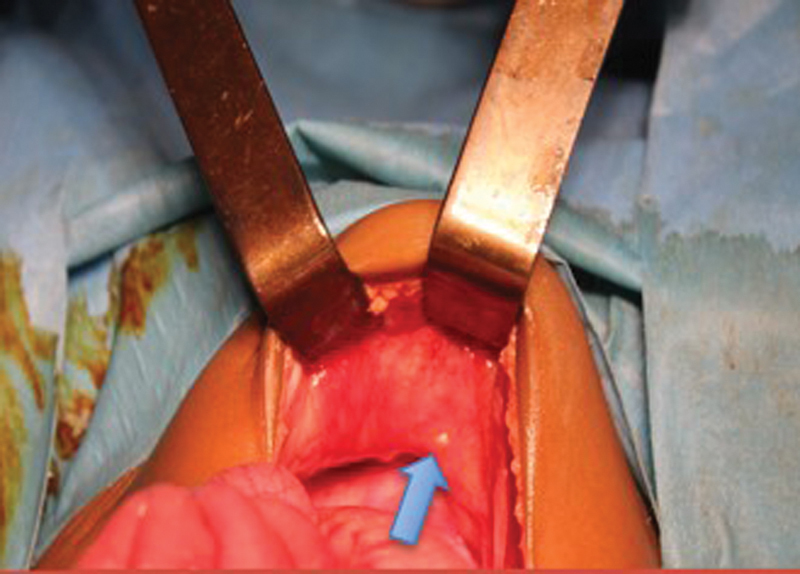
Granulations on the abdominal wall (see arrow).

**Fig. 3:**
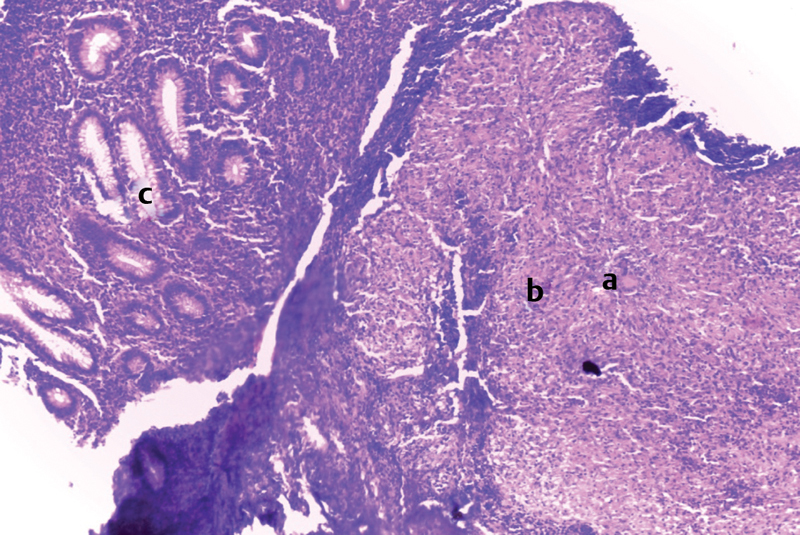
Appendiceal mucosa presenting an inflammatory granuloma involving tuberculous follicle in the submucosa. ( **a** ) Giant cells. ( **b** ) Epithelïoid cells. ( **c** ) Appendiceal mucosa.

**Fig. 4:**
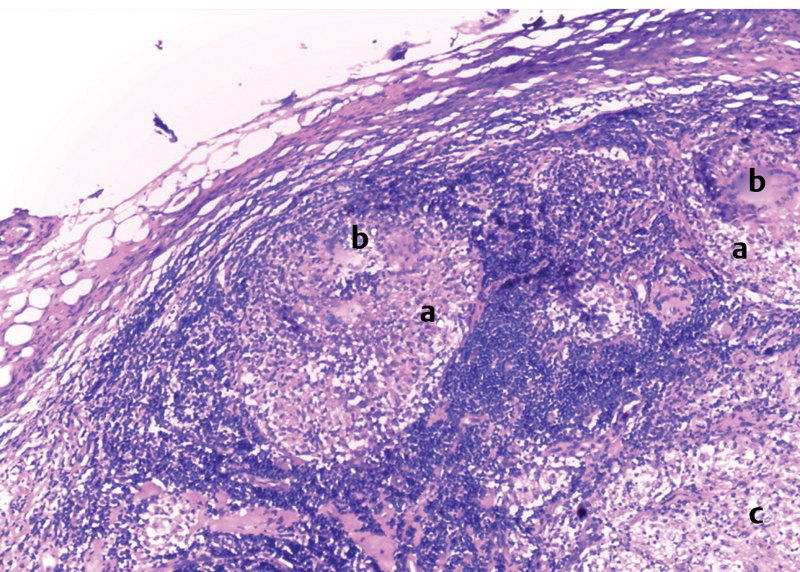
Ganglionic parenchyma site of inflammatory granulomas. ( **a** ) Epithelïoid cells. ( **b** ) Giant cells in concentric arrangement surrounded by lymphocytes. ( **c** ) Signs of starting necrosis.

A 6-month-old female infant (6000 g; 3rd percentile) with an unremarkable medical history was transferred to our pediatric ward because of postprandial vomiting for 5 days. On clinical examination, she was subfebrile (38°C) and had a distended but soft abdomen. No mass was palpable. Abdominal ultrasound suggested the possibility of intussusception. As an enema to reduce intussusception was no therapeutic option at our center, the abdomen was surgically explored. On laparotomy, blood-stained but clear ascites was found as well as intestinal adhesions. Moreover, we saw diffuse granulations, mesenteric lymphadenopathy, and ischemia of the terminal ileum ( Figs. 1 and 2 ). Peritoneal cultures were harvested, appendectomy was performed, and the intestinal lymph nodes were biopsied. Postoperatively, tuberculin intradermal reaction (IDRt) was positive at 9 mm and cultures grew tuberculous contagium. The Anti-TB treatment administered was: rifampicin, isoniazid, pyrazinamide, ethambutol (RHZE) for 2 months, then RH the following 4 months. This regimen was applied before receiving the biopsy results which showed tuberculoid follicular lesions ( Figs. 3 and 4 ). The patient was declared cured after this treatment. She had no abdominal symptoms after 2 years of follow-up.

### Case 2

An 8-month-old (7000 g; 3rd percentile) boy from Mauritania with no particular pathological history was transferred from a health center near the Senegal–Mauritanian border for the management of peritonitis. The clinical picture had evolved for 15 days, with the appearance of abdominal bloating associated with vomiting and cessation of bowel movements. On clinical examination, he had a temperature of 38°C, and was tachycardic at 148 beats per minute. He showed nutritional edema and flank dullness. His abdomen was distended and had peritonitis on palpation. Therefore, the boy was taken to the operating room. Surgical exploration revealed clear ascites associated with small intestinal granulations and adhesions. We performed adhesiolysis, biopsies, and drainage of the peritoneal cavity. Postoperatively, GeneXpert tests were performed on the peritoneal fluid and the IDRt was negative. Anatomopathological examination of the operative specimens revealed the presence of caseous necrosis compatible with peritoneal TB. The medical treatment and the evolution were identical to Case 1.

## Discussion

The most common digestive tract localization of pediatric TB is the peritoneal serosa. 6 Malnutrition is the leading predisposition for the reactivation of a latent peritoneal focus. 2 This comorbidity was noted in our second patient. In our center, the leading cause of an acute abdomen in infants with low-grade fever and disturbances in bowel mobility is intussusception. It is difficult to diagnose peritoneal TB because of clinical polymorphism and the absence of specific radiological findings. In infants, this diagnostic challenge is more obvious because of the high prevalence of digestive tract infections with infectious organisms other than TB. Febrile ascites is the most common clinical manifestation. The diagnostic difficulties of peritoneal TB have been reported by several authors. 7 8 9 The definitive diagnosis of abdominal TB is made by histology of bioptic specimens. 4 7 Some authors recommend to obtain these biopsies by laparoscopy which allows good exploration of the peritoneal cavity and a sensitivity close to 100%. 4 5 6 7 8 9 However, the long time spent to obtain the results of the pathological examination justifies the use of a certain number of diagnostic arguments (clear ascites, granulations, IDRt, GeneXpert) and allow us to start the treatment before the histology results. IDRt does not provide the diagnosis of peritoneal TB. 4 5 However, combined with other clinical and epidemiological parameters, it can be an important element in decreasing the treatment period. In our first patient, the discovery of diffuse granulations throughout the abdomen associated with a clear ascites, the positive cultures for tuberculous contagium, in combination with a positive IDRt allowed to start the medical treatment before the results of the anatomic pathological examination were available. The accuracy GeneXpert is affected by the sampling times and the conditions during transport. The sensitivity has been reported to be as low as 60 to 80%. 10 The first patient was operated at night and the laboratory that performs this examination is not open at night which explains why the test was not performed in this patient. In the second patient the geneXpert is negative because the sensitivity of this test is not 100%.

## Conclusion

Peritoneal TB is rare in infants, and diagnosis can be challenging. In children with abdominal pain, low-grade fever, and ascites, this differential diagnosis should be taken into consideration, especially in endemic arias. Medical treatment is usually effective.

## Notes

New Insights and the Importance for the Pediatric Surgeon Pectus excavatum (PE) is the most common malformation of the anterior chest wall. The use of minimally invasive repair for pectus excavatum in extremely severe cases of PE is still controversial and adequate severity assessment indexes should be ensued.
